# Intercostal Nerve Cryoablation or Epidural Analgesia for Multimodal Pain Management after the Nuss Procedure: A Cohort Study

**DOI:** 10.1055/a-2249-7588

**Published:** 2024-02-16

**Authors:** Hendrik van Braak, Sjoerd A. de Beer, Justin R. de Jong, Markus F. Stevens, Gijsbert Musters, Sander Zwaveling, Matthijs W. N. Oomen, Wendeline Van der Made, Egbert Krug, L.W. Ernest van Heurn

**Affiliations:** 1 Department of Pediatric Surgery, Amsterdam UMC Locatie AMC, AZ, Amsterdam, Noord-Holland, The Netherlands; 2 Department of Anesthesiology, Amsterdam UMC Locatie AMC, Amsterdam, North Holland, The Netherlands; 3 Department of Gastrointestinal Surgery and Surgical Oncology, Erasmus MC, Rotterdam, Zuid-Holland, The Netherlands; 4 Department of Surgery, Leiden Universitair Medisch Centrum, Leiden, Zuid-Holland, The Netherlands

**Keywords:** Nuss procedure, pectus excavatum, intercostal nerve cryoablation, continuous epidural analgesia

## Abstract

**Background** Nuss procedure for pectus excavatum is a minimally invasive, but painful procedure. Recently, intercostal nerve cryoablation has been introduced as a pain management technique.

**Materials and Methods** In this cohort study, we compared the efficacy of multimodal pain management strategies in children undergoing a Nuss procedure. The effectiveness of intercostal nerve cryoablation combined with patient-controlled systemic opioid analgesia (PCA) was compared with continuous epidural analgesia (CEA) combined with PCA. The study was conducted between January 2019 and July 2022. Primary outcome was length of stay (LOS), and secondary outcomes were operation room time, postoperative pain, opioid consumption, and gabapentin use.

**Results** Sixty-six consecutive patients were included, 33 patients in each group. The cryoablation group exhibited lower Numeric Rating Scale (NRS) pain scores on postoperative day 1 and 2 ( *p* = 0.002, *p* = 0.001) and a shorter LOS (3 vs. 6 days ( *p* < 0.001). Cryoablation resulted in less patients requiring opioids at discharge (30.3 vs. 97.0%; *p* < 0.001) and 1 week after surgery (6.1 vs. 45.4%; *p* < 0.001)). In the CEA group, gabapentin use was more prevalent (78.8 vs. 18.2%; *p* < 0.001) and the operation room time was shorter (119.4 vs. 135.0 minutes; *p* < .010). No neuropathic pain was reported.

**Conclusions** Intercostal nerve cryoablation is a superior analgesic method compared with CEA, with reduced LOS, opioid use, and NRS pain scores. The prophylactic use of gabapentin is redundant.

## Introduction

Pectus excavatum (PE) is a common chest wall deformity that results from sternal displacement due to rib cartilage overgrowth. The incidence is estimated at 1 in 40 to 400 individuals. 1 Conventional treatment for PE is minimally invasive repair of pectus excavatum, known as the Nuss procedure. 2

This procedure causes significant postoperative pain, often resulting in an extended length of hospital stay (LOS) and increased opioid use. In most hospitals, thoracic continuous epidural analgesia (CEA) is the primary method of pain management after the Nuss procedure. 3 Because increased infection risk limits its use to 3 days, CEA is often supplemented with patient-controlled systemic opioid analgesia (PCA).

Thoracoscopic intercostal nerve cryoablation offers an alternative postoperative pain management approach. Alone or combined with the standard of care in multimodal pain protocols, it reduces postoperative opioid use and LOS. 2 4 5 However, uncertainties remain regarding long-term complications, particularly neuropathic pain (for which prophylactic gabapentin is used). Although there is abundance of studies, pain scores in the only randomized controlled trial did not differ between cryoanalgesia and CEA. 2 Furthermore, most studies do not report pain scores or the incidence of opioid medication at discharge or later.

We share our experience with intercostal nerve cryoablation and CEA in the Amsterdam Pectus Center, with special focus on postoperative pain scores and the need for prophylactic gabapentin use.

## Materials and Methods

### Study Population and Design

We performed a cohort study of patients (<18 years) undergoing a Nuss procedure at the Amsterdam Pectus Center and Maastricht University Medical Center between January 2019 and July 2022.

The prospectively included cryoablation cohort consisted of the first patients undergoing the Nuss procedure during our trial period with cryoablation and PCA. The retrospectively included CEA cohort consisted of the last patients undergoing the Nuss procedure with CEA and PCA. Both evenly sized cohorts consisted of consecutive patients. No case-matching was performed. The number of patients undergoing the Nuss procedure during the study period determined the sample size.

Patients received CEA combined with PCA for 3/4 days, or intraoperative bilateral thoracoscopic intercostal nerve cryoablation with PCA. If necessary, additional pain treatment was administered according to our pain protocol. No other intraoperative nerve blocks, preoperative analgesics, or anxiolytics were given.

### Data Extraction

Patient and treatment characteristics were retrospectively obtained from patient records. This included analysis of operative reports, radiology reports, and patient charts to gather information on patient demographics, primary treatment characteristics, operative technique, hospital stay, and outpatient follow-up.

Numeric Rating Scale (NRS) pain scores (average score of up to three daily NRS pain scores) were recorded on day 0 (day of surgery), 1 day postsurgery, and 2 days postsurgery. Opioid prescriptions were documented at discharge. One week after discharge, patients were contacted via telephone to gather information on opioid use. After 6 weeks, the patients visited the outpatient clinic for an evaluation of current medication and opioid use. Minimum follow-up period was 12 months.

### Outcomes

Primary outcome was LOS. Secondary outcomes were opioid consumption, NRS pain scores, use of PCA, operation room time, and gabapentin use.

### Statistical Analysis

Descriptive measurements were utilized to characterize the study population. The Shapiro–Wilk test was employed to determine parametricity of continuous variables. Nominal or categorical data were analyzed using Fisher's exact test or the chi-squared test. Continuous variables were analyzed using the Mann–Whitney U test or the independent sample *t* -test. Statistical significance was set at a probability value of less than 0.05. Data were analyzed using IBM SPSS Statistics version 28.0.

## Results

### Patient Characteristics

All patients undergoing the Nuss procedure were examined for eligibility, and all ( *n* = 66) patients in the study period were confirmed to be eligible and included in the study. None of the patients was lost to follow-up. Thirty-three patients were included in each cohort. Of all patients, 84.8% were male ( *n* = 56/66) and median age was 16.0 years. Most patients received one Nuss bar (84.8%, *n* = 56/66). Median follow-up was 35.2 (interquartile range [IQR]: 17.0–44.9]) months. No readmissions were reported. Baseline characteristics are described in Table 1 , and perioperative and postoperative characteristics and outcomes are described in Table 2 .

**Table 1:** Patient characteristics

	All ( *n* = 66)	CEA ( *n* = 33)	Intercostal nerve cryoablation ( *n* = 33)
Sex b			
Male	56 (84.8)	27 (81.8)	29 (87.9)
Female	10 (15.2)	6 (18.2)	4 (12.1)
Age a	16.0 (IQR: 15.0–17.0])	16.0 (IQR: 14.0–16.0])	16.0 (IQR: 16.0–17.0)
Follow-up (months) a	35.2 (IQR: 17.0–44.9)	44.8 (IQR: 42.3–47.4)	17.0 (IQR: 15.4–32.9)
Severity of pectus excavatum b			
Light	11 (16.6)	3 (9.1)	8 (24.2)
Moderate	45 (68.2)	23 (69.7)	22 (66.7)
Severe	10 (15.2)	7 (21.2)	3 (9.1)
Nuss bar(s) b			
One	56 (84.8)	24 (72.7)	32 (97.0)
Two	10 (15.2)	9 (27.3)	1 (3.0)

Abbreviations: CEA, continuous epidural analgesia; IQR, interquartile range; N/n, number; *p* -value, probability value.

a Nonparametric continuous variables, expressed as median (IQR).

b Data displayed as *n* (%).

**Table 2:** Duration of surgery, pain scores, length of hospital stay, opioid consumption and complications

	CEA ( *n* = 33)	Intercostal nerve cryoablation ( *n* = 33)	*p* -Value
Operation room time (min.) a	119.4 ± 22.7	135.0 ± 31.3	0.010
Postoperative VAS score			
Day 0 a	3.1 ± 1.9	2.4 ± 1.8	0.133
Day 1 a	4.2 ± 1.9	2.9 ± 1.7	0.002
Day 2	4.0 (IQR: 2.5–5.0])	2.0 (IQR: 2.0–3.0)	0.001
Stop of PCA (days after surgery) a	4.1 ± 1.9	1.6 ± 0.9	<0.001
LOS (days)	6.0 (IQR: 5.0–8.0)	3.0 (IQR: 2.0–4.0)	<0.001
Opioid use b			
At discharge	32 (97.0)	10 (30.3)	<0.001
At discharge + 1 week	15 (45.5)	2 (6.1)	<0.001
At discharge + 6 weeks	1 (3.0)	1 (3.0)	1.000
Gabapentin use b	26 (78.8)	6 (18.2)	<0.001

Abbreviations: CEA, continuous epidural analgesia; IQR, interquartile range; LOS, length of hospital stay; min., minutes, N/n, number; PCA, patient-controlled systemic opioid analgesia; *p* -value, probability value; VAS, visual analogue scale.

a Parametric variables; expressed as mean ± standard deviation; all remaining continuous variables are nonparametric and expressed as median (IQR).

b Data displayed as *n* (%).

### Operation Room Time, Pain Management, and Opioid Consumption

Mean operation room time was 15.6 (95% confidence interval [CI]: −2.2 to −29.0) minutes shorter for the CEA group, *p* < 0.010) compared with the cryoablation group. There was no significant difference in operation room time between the Nuss procedure with one or two bars (127.0 vs. 132.0, *p* = 0.365).

NRS pain scores at day 1 and 2 were lower in the cryoablation group (respectively −1.3 (95% CI: −2.2 to −0.4), *p* = 0.002 and −2.0 (IQR: −.5 to −3.0), *p* = 0.001). Use of gabapentin (78.8%, *n* = 26/33 vs. 18.2%, *n* = 6/33) was higher in the CEA group ( *p* < 0.001).

**Fig. 1:**
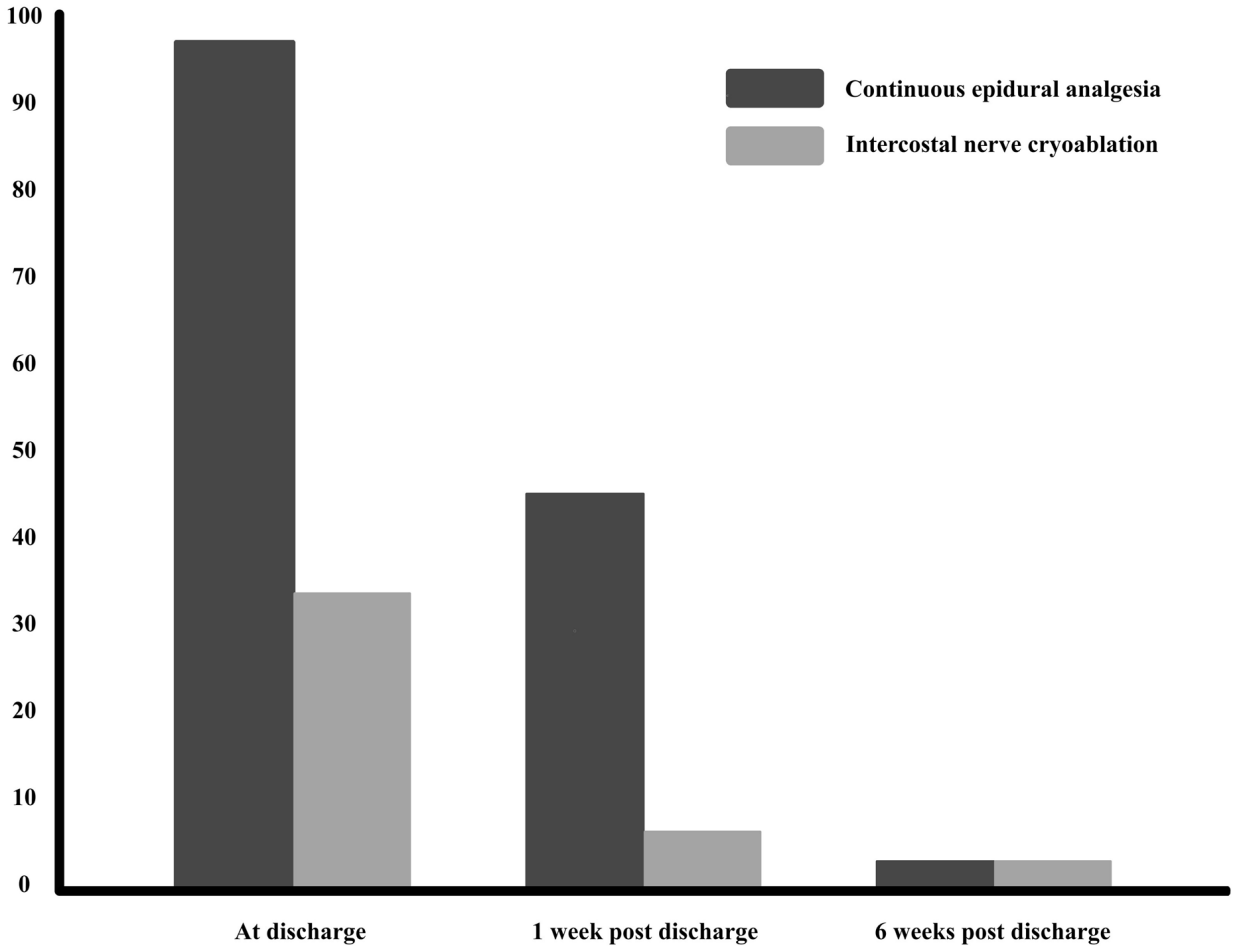
Comparison of opioid use in both treatment groups y-axis: percentage of patients using opioids.

Differences in opioid use at discharge, after 1 and after 6 weeks are visualized in Fig. 1 . Fewer patients used opioids at discharge ( *n* = 10/33, 30.3% vs. *n* = 32/33, 97.0%; *p* < 0.001)) and 1 week after surgery ( *n* = 2/33, 6.1% vs. *n* = 15/33, 45.4%; *p* < 0.001)) in the cryoablation group. After 6 weeks two patients still used opioids, one in each group. The use of PCA after surgery was shorter in the cryoablation group (1.6 vs. 4.1 days, *p* < 0.001).

### LOS

**Fig. 2:**
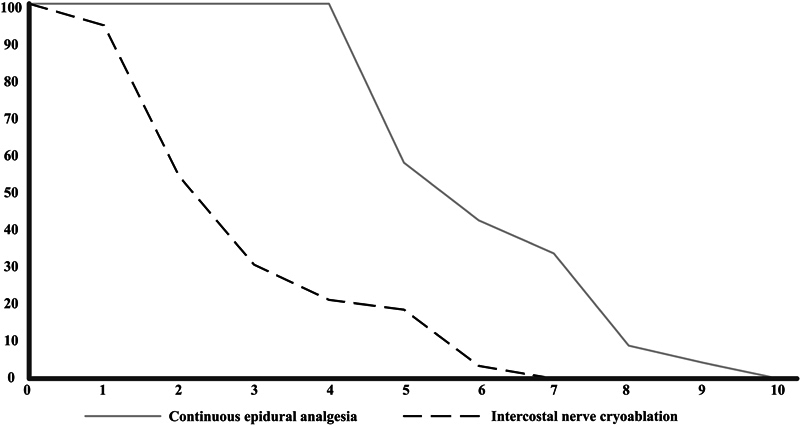
Comparison of length of hospital stay in both treatment groups y-axis: percentage of patients still hospitalized, x-axis: days.

Median LOS was 3 days shorter in the cryoablation group ( Fig. 2 ). In the cryoablation group, seven patients stayed for 5 days or longer (18 in the CEA group). In two cases, the prolonged stay was related to the cryoablation procedure (large bilateral pneumonia [ *n* = 1], pain due to unilateral cryoablation [ *n* = 1]). Other reasons for prolonged hospital stay were social problems ( *n* = 2), opioid related gastrointestinal problems ( *n* = 2), and persistent pain ( *n* = 1).

## Discussion

Intercostal nerve cryoablation combined with PCA as multimodal pain management for Nuss procedure leads to clinically relevant reduction in pain levels compared with the combination of CEA with PCA. This leads to reduced LOS and a reduction in the use of opioids postoperatively, at discharge and 1 week after discharge.

### Operation Room Time

This study shows a comparable operation room time between CEA and cryoablation, similar to the only other study reporting on this matter. 3 Since epidural placement is essential in the Nuss procedure, we consider operation room time more relevant than surgery time when comparing both treatments. Our findings put the argument that cryoablation is time-intensive, and consequently more expensive compared with CEA, into perspective.

### Postoperative Pain

Directly postoperative (day 0) the NRS pain scores were similar between both groups, which is consistent with current literature. 2 6 7 Only one study demonstrated an advantage of cryoablation. 8 This was likely the effect of perioperatively titrated opioids and anesthetics. We reported statistically significant and clinically relevant differences in NRS pain scores on day 1 and 2 between the groups, which have not yet been described before.

### Opioid Use

Postoperative opioid use is defined in different ways. Most studies use total oral morphine milligram equivalents to assess differences in opioid use. We measured the percentage of patients using opioids or PCA at specific time points. Perez Holguin et al used both methods and obtained similar results as ours (discontinuation of PCA after 1 day vs. after 4 days), also showing a difference in percentage of patients requiring narcotics on discharge (100 vs. 54.8%). 5 We achieved even better results at discharge, and further demonstrated that opioid usage after 1 week remains lower in the cryoablation group. This is a promising result, which has not yet been described before.

### LOS

The LOS was 3 days shorter in the cryoablation group, which is similar to the literature (LOS varying from −2 to −3 days). 3 4 5 8

Since cryoablation was implemented after years of providing extensive multimodal pain therapy, requiring long hospitalization, patients in the cryoablation cohort might have been hospitalized for too long. An analgesic regimen that further reduces opioid use may reduce LOS even further. 3 This suggests the possibility of same-day discharge, as supported by promising results from a pilot study. 9

### Neuropathic Pain and Gabapentin Use

We reported no patients with neuropathic pain, which is consistent with current literature. 10 Despite the low incidence of neuropathic pain in adolescents, standard prescription of prophylactic gabapentin and/or muscle relaxants during hospitalization and/or after discharge is described in two studies. 3 6 Others used gabapentin only on indication. 2 5 However, all studies reported a low incidence of neuropathic pain. In our population gabapentin (300 mg, twice daily for 5 days) was prescribed postoperatively in all patients weighing more than 40 kg in the CEA group and in six patients in the cryoablation group.

### Limitations

The characteristics of the study and the small sample size limits the generalizability of the results. However, there is a large difference between both groups, both in use of analgesics and LOS.

## Conclusion

We conclude that intercostal nerve cryoablation is a superior analgesic method compared with CEA, with reduced LOS, opioid usage, and NRS pain scores. We recommend against the routine use of gabapentin and leave its use for specific neuropathic pain.
